# ToMAS: Torus-based secure multi-factor biometric authentication system

**DOI:** 10.1016/j.csbj.2025.10.042

**Published:** 2025-10-23

**Authors:** Mi Yeon Hong, Kang Hoon Lee, Ji Hyuk Jung, Ji Won Yoon

**Affiliations:** Graduate School of Cybersecurity, Korea University, 405, Science Library, Anamro 145, Seoul, 02841, Seoul, South Korea

**Keywords:** Fully homomorphic encryption, Privacy-preserving, Biometric authentication

## Abstract

Biometric authentication has emerged as a convenient method for identity verification, but its widespread adoption raises serious privacy concerns. In this paper, we propose a Torus-based secure Multi-factor biometric Authentication System (ToMAS) that addresses these concerns by securing both the enrollment and authentication phases through cryptographic protocols. ToMAS adopts a multi-factor approach using both physiological biometric traits and password-derived secrets, and leverages fully homomorphic encryption (FHE) to perform computations on encrypted data without revealing sensitive information.

To reduce overhead and improve efficiency, we introduce a ciphertext packing method and a modified bootstrapping technique for secure Hamming distance evaluation. Our protocol is analyzed against active adversaries. Experiments show that a 9600-bit binary biometric template can be encrypted into an 82KB ciphertext, and the Hamming distance between encrypted templates is computed in under one second on a standard AMD Ryzen Threadripper Pro CPU, with no loss of accuracy. ToMAS offers an efficient and scalable solution suitable for large-scale biometric authentication scenarios.

## Graphical Abstract

**Figure:**
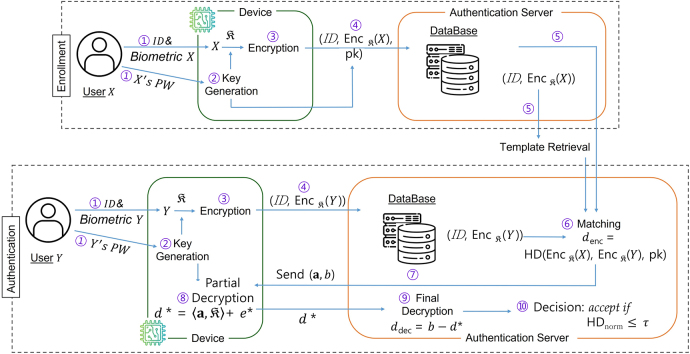

## 1 Introduction

### 1.1 Problem

User authentication encompasses a wide range of human–machine interactions and is typically categorized into three types based on (i) knowledge, (ii) possession, or (iii) biometrics. Biometrics involve individuals’ unique physical, physiological, or behavioral traits, such as fingerprints, faces, irises, veins, voices, and handwriting. Unlike knowledge- and possession-based authentication, biometrics rely on inherent personal characteristics, reducing risks associated with theft or loss. As a result, biometric authentication has rapidly proliferated across diverse commercial applications.

However, increasing biometric prevalence raises significant privacy concerns, including potential leaks, forgeries, or alterations. Because of biometric data’s intrinsic and sensitive nature, compromised templates cannot be altered or reset, which necessitates enhanced security precautions.

Authentication typically consists of two phases: enrollment and authentication [1]. During enrollment, the user’s biometric template is securely stored along with their identification (ID) and password (PW). During authentication, a freshly submitted biometric template is compared against the stored template, and the user is authenticated according to a predefined similarity measure. The biometric template’s concise yet highly sensitive nature exacerbates security and privacy challenges across these phases.

Any biometric exposure can facilitate attacks, such as hill-climbing [2] or replay attacks [3] using forged templates, undermining system security. Utilizing biometrics in unsupervised environments amplifies these vulnerabilities, as discussed in studies by Alaswad et al. [4] and Uludag et al. [5]. Furthermore, the inherent irreplaceability of biometric traits means compromised templates cannot be re-issued.

Recognizing these risks, regulations such as GDPR explicitly classify biometrics as personal information and outline detailed protection guidelines, and international standards such as ISO/IEC 24745 provide specific guidance on biometric information protection. However, despite extensive research into privacy-preserving biometric authentication [6], [7], [8], [9], [10], recent studies still highlight persistent security challenges [11], [12], [13], [14], [15].

Hence, a crucial challenge remains: developing practical biometric authentication protocols that robustly preserve privacy without sacrificing authentication accuracy.

### 1.2 Our contribution

We address the privacy and performance limitations of existing biometric authentication systems by introducing a user-centric, efficient, and secure solution called ToMAS. Our main contributions are as follows:

**– Practical user-centric system design:** We propose a secure biometric authentication system that eliminates reliance on trusting a central server with plaintext biometrics. Traditional *server-centric* methods require users to trust the provider with their biometric data, making privacy vulnerable if the service provider acts maliciously or is compromised. Cross-matching across databases can also reveal a user’s identity across services, severely impacting privacy.

To address this, Zhou and Ren [16] introduced a *user-centric* system where encrypted templates are shared with providers. While it improves privacy, their method suffers from scalability issues as computational cost increases rapidly with template size.

In this paper, we propose an efficient *user-centric* authentication system suitable for large-scale biometric templates. The core idea of the system is that the user directly provides the authentication factors. We employ a multi-factor authentication approach that combines biometric data (as the first factor) and a password-derived key (as the second, knowledge-based factor). The similarity score is partially decrypted by the user using their secret key and then sent to the server. The server performs only the final decryption, ensuring that sensitive biometric information remains protected during the authentication process.

**– Practical ciphertext-level defense against active attacks:** Although several works have analyzed threats to biometric systems [13], [17], [18], many assume passive attackers or rely on the existence of a trusted channel between protocol entities, thereby only implicitly addressing security against active attackers. While THRIVE [19] addresses malicious environments, it relies on a semi-honest model and complex key management. ToMAS introduces a practical replay-binding countermeasure for TFHE-based matching using timestamp-derived FHE operations. By embedding one-time hash-derived randomness into encrypted templates, replay attempts result in invalid computations. We formally present a privacy-preserving authentication protocol with verifiable security under active attack scenarios.

**– High-performance FHE-based biometric authentication:** When a template vector is encrypted bit-by-bit, the time complexity and computational cost of calculating the Hamming distance significantly increase because a large number of ciphertexts must be handled for a single template. In this paper, we apply a method based on the fast fully homomorphic encryption scheme over the torus (TFHE) [20], which dramatically reduces memory usage by packing multiple bits into a single ciphertext using a ring-based message space [21]. Compared to conventional *Learning With Errors* (LWE)-based bitwise encryption, this packing can save up to $\frac{n+1}{k+1}$ times of storage, where n and k are positive integers denoting the dimensions of LWE and Torus LWE (TLWE) spaces, respectively, and n is usually significantly larger than k.

Our approach to calculating the Hamming distance is based on modified binary gate operations that evaluate Boolean circuits over encrypted templates. As a result, the template is mapped to $\frac{1}{q}Z_{q}$ within the torus T, enabling efficient and secure computation without any loss.

While cryptographic approaches such as Secure Multi-Party Computation (MPC), Homomorphic Encryption (HE), and Inner-Product Encryption (IPE) offer privacy, only FHE supports arbitrary computation over encrypted data. However, bootstrapping remains a major bottleneck, and libraries like Cheon-Kim-Kim-Song (CKKS) [22] and Fan–Vercauteren (FV) [23] are not optimized for gate-level logic required in biometric comparisons. TFHE enables accurate computation through binary gate operations, but suffers from per-bit latency. ToMAS mitigates these limitations by combining packing and efficient bootstrapping.

We implemented ToMAS on an AMD Ryzen Threadripper Pro (3.6 GHz, 64 GB RAM). The Hamming distance for a 9,600-bit encrypted template was computed in 0.878 s, 0.941 s, and 0.996 s under security levels λ=80, 112, and 128, respectively. Compared to prior systems, ToMAS demonstrates well-defined security properties under the stated assumptions in the presence of active attacks, efficient scalability to large biometric data, and superior performance. To the best of our knowledge, ToMAS represents one of the fastest and most practical FHE-based biometric authentication systems to date.

## 2 Related work

### 2.1 A privacy-preserving multi-factor authentication

Multi-factor authentication (MFA) strengthens identity verification by requiring multiple independent factors to access services. To enhance privacy, Bhargav-Spantzel et al. [24] introduced protocols using zero-knowledge proof (ZKP), where authentication factors include credit card numbers (CCNs) or social security numbers (SSNs).

As mobile computing rapidly expanded, Soares et al. [25] highlighted the need for more robust MFA and introduced a scheme combining traditional login with a one-time password (OTP). Liu et al. [26] proposed MACA, and Acar et al. [27] introduced PINTA—both systems using passwords and hybrid behavioral profiles. They applied fuzzy hashing and FHE to protect sensitive data from server exposure. Anakath et al. [28] further enhanced privacy in cloud environments by eliminating the need for extra physical devices.

Although all of the aforementioned studies are labeled as MFA, they actually address two factors. Several works of literature can be found in three-factor authentication studies [29], [30], [31], all of which deal with a smart card, password, and biometric characteristics. These protocols achieve a higher security guarantee and preserve user privacy.

### 2.2 Homomorphic encryption schemes for biometric authentication

Recently, to guarantee the complete privacy of biometric data, research on HE techniques for secure matching algorithms has been conducted.

Cheon et al. [32] proposed a scheme using somewhat homomorphic encryption (SHE) [33], [34] and a one-time message authentication code (OTM). Barrero et al. [35] developed a multi-biometric protection method based on Paillier encryption [36], fusing signature and fingerprint data at different processing levels. Yang et al. [37] applied a modified minutiae pair representation to Paillier-based fingerprint authentication.

Song et al. [38] implemented an iris authentication system using the FV scheme and Microsoft’s SEAL library [39], where the server completes matching via OTM. Morampudi et al. [40] used FHE to compare encrypted iris templates and extended their work into SvaS [41], a secure and verifiable classifier. Pradel et al. [13] demonstrated a TFHE-based privacy-preserving authentication protocol, validating the practicality of fully homomorphic protection for biometric data.

More recently, Choi et al. proposed Blind-Touch [42], a CKKS-based distributed neural network inference framework for fingerprint authentication, achieving real-time inference but with limited accuracy for 1:N identification. To address these limitations, Blind-Match [43] introduced a CKKS-optimized cosine similarity algorithm, enabling efficient 1:N biometric matching with Rank-1 accuracies of over 99 % on both face and fingerprint datasets. Furthermore, AMB-FHE [44] demonstrated adaptive multi-biometric fusion under CKKS, combining iris and fingerprint features. While achieving an EER of 0.08 %, the heavy cost of rotation operations highlights the trade-off between accuracy and efficiency.

Overall, prior HE-based biometric authentication schemes can be divided into two lines. CKKS-based approaches offer high throughput and efficient approximate computation, but their reliance on floating-point arithmetic introduces approximation errors and additional costs when scaling to large template sizes. By contrast, TFHE enables exact gate-level operations, which preserve matching accuracy without approximation, but suffer from per-bit latency. This distinction underscores the accuracy–efficiency trade-off between approximate (CKKS) and exact (TFHE) methods, and motivates the design of systems such as ToMAS that aim to combine efficiency with exact computation.

## 3 Preliminaries

### 3.1 Fully homomorphic encryption (FHE)

Fully Homomorphic Encryption (FHE) enables arbitrary operations on encrypted data without decryption. For operations ∙ and ⋆, FHE is defined when the following holds:

$$\begin{array}{l} \mathrm{Dec}\;[\mathrm{Enc}(x_{1})∙\mathrm{Enc}(x_{2})]=\mathrm{Dec}\;[\mathrm{Enc}(x_{1}⋆x_{2})], \end{array}$$

where $x_{1},x_{2}$ are plaintexts, and Enc,Dec denote encryption and decryption.

### 3.2 Glossary of notation

Table 1 summarizes the main mathematical symbols and parameters used throughout this paper for consistency.

**Table 1:** Glossary of notation.

Symbol	Description
B:={0,1}	Binary space
$Z_{q}:=Z/qZ$	Plaintext space (modulus q, power of two)
T:=R/Z	Torus (real numbers mod 1)
$T_{N}[X]:=T[X]/(X^{N}+1)$	Ring of torus polynomials
$K\in B^{n}$	LWE secret key (binary vector of dimension n)
$K\in B_{N}{\left[X\right]}^{k}$	TLWE secret key (polynomial vector of length k)
sk:=(K,K)	TFHE secret key
pk:=(BK,ksk)	Evaluation key (bootstrapping key and key-switching key)
a,b	LWE vector and scalar in $T^{n}\times T$
⟨a,b⟩	Inner product
ct,CT	LWE / TLWE ciphertexts
$\varphi_{K}(\mathrm{ct})$	LWE phase b−⟨a,K⟩
$\varphi_{K}(\mathrm{CT})$	TLWE phase B−⟨A,K⟩
μ	Plaintext message in LWE/TLWE encryption
$\mu_{\text{LUT}}$	Lookup-table coefficient (e.g., $\frac{1}{2q}$ for XOR)
$M:=\{-\frac{1}{8},\frac{1}{8}\}$	Standard Boolean message space in TFHE
$M^{\prime }:=\{0,\frac{1}{q}\}$	Transformed message space for accumulated XORs
HD(X,Y)	Hamming distance between binary vectors
n	LWE dimension
N	Polynomial degree for TLWE (power of two)
k	TLWE dimension (number of polynomials)
σ	Standard deviation of error distribution
χ	Discrete Gaussian error distribution with σ
$B,B_{g},B_{\text{ks}}$	Decomposition bases for TGSW / key-switching
$\ell ,\ell_{\text{ks}}$	Decomposition lengths for TGSW / key-switching
λ	Security parameter (bits)
q	Plaintext modulus

### 3.3 Overview of TFHE

Chillotti et al. [20] proposed the TFHE scheme, built on the LWE problem and its ring variant (RLWE). It extends FHEW [45] to support binary gate-level operations on encrypted messages. TFHE works over T and the polynomial ring $T_{N}[X]$.

*Ciphertext types*.

• **LWE:** ct=(a,b) with b=⟨a,K⟩+μ+e, e←χ.
• **TLWE:** CT=(A,B) with B=⟨A,K⟩+μ+e.
• **TGSW:** Matrix form of TLWE used to encode the bootstrapping key.

#### 3.3.1 Bootstrapping

To reduce noise and allow unbounded depth, bootstrapping consists of three steps: BlindRotate → SampleExtract → KeySwitch. Input messages in T are mapped to $Z_{2\;N}$ via modulus switching, e.g.,

$$\begin{array}{l} 0.25\in T\;\mapsto \;\lfloor 0.25\cdot 2\;N\rfloor \in Z_{2\;N}. \end{array}$$

### 3.4 TFHE algorithms

• **KeyGen** (λ)**:** Generate K,K and evaluation key pk=(BK,ksk).
• **Enc** (μ,K)**:** Sample a,e; output ct=(a,⟨a,K⟩+μ+e).
• **Eval (XOR/AND):**
  $$\begin{array}{l} \mathrm{HE}\_\mathrm{AND}({\mathrm{ct}}_{1},{\mathrm{ct}}_{2}):=\mathrm{Bootstrap}\left(\left(0,-\frac{1}{8}\right)+{\mathrm{ct}}_{1}+{\mathrm{ct}}_{2}\right). \end{array}$$
  Offset $-\frac{1}{8}$ maintains alignment with $M=\{-\frac{1}{8},\frac{1}{8}\}$.
• **Dec** (ct,K)**:** Compute ϕ=(b−⟨a,K⟩)mod1=μ+e, then round to nearest in M.

## 4 Proposed approach

**Fig. 1:**
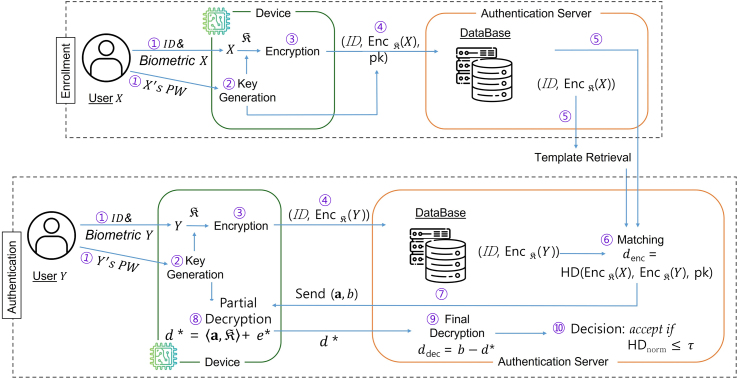
Overall workflow of ToMAS: end-to-end encrypted enrollment and authentication involving U, D, DB, and AS.

To realize fully homomorphic biometric authentication, we design an efficient framework that integrates ciphertext packing and modified bootstrapping as the core mechanisms of ToMAS. The system involves four entities: the **user** (U), the **device** (D), the **database** (DB), and the **authentication server** (AS). Here, U interacts with D for biometric capture and PW input; DB securely stores encrypted templates; and AS performs encrypted verification using the evaluation keys, as illustrated in Fig. 1.

### 4.1 Key generation

ToMAS adopts multi-factor authentication combining a biometric factor and a password-derived cryptographic key. This process applies to both the enrollment and authentication phases.

①  When the U scans a biometric through the D and inputs a password PW, ②  the PW is not used directly as the TFHE secret key. Instead, it is processed together with a user-specific salt via a password-based key derivation function (PBKDF), such as Argon2id, configured with high memory and iteration costs to yield a 256-bit seed. The seed is expanded by a cryptographically secure pseudorandom generator (CSPRNG) into the TFHE secret key $K\in B^{n}$. Since pseudorandom generator (PRG) outputs are indistinguishable from uniform, the derived key maintains the distributional requirements of TFHE (as depicted in Table 2).

**Table 2:** Recommended Argon2id parameters for password-derived key generation.

Parameter	Description	Recommended value
M	Memory cost	≥64 MB (desktop) / ≥256 MB (server)
t	Time cost	3–5 iterations
p	Parallelism	1–4 threads
s	Salt length	128 bits
h	Output length	256 bits

③  A biometric template is extracted via preprocessing and feature encoding. Both during enrollment and authentication, the user encrypts the biometric templates X and Y under the same K. The same K can be deterministically reconstructed from PW; the salt prevents pre-computation attacks, PBKDF increases brute-force cost, and the CSPRNG preserves key uniformity. A strong password policy (≥12 characters, mixed classes) is required to ensure sufficient entropy.

### 4.2 Encryption with message packing

**Fig. 2:**
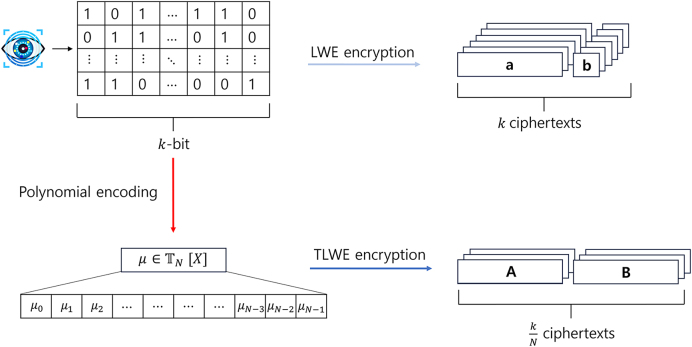
Packing binary biometric templates into a TLWE ciphertext.

The U generates (K,K,BK,ksk). Each biometric template is encrypted as a TLWE ciphertext to enable polynomial-level packing:

$$\begin{array}{l} \mu =\sum_{i=0}^{N-1}\mu_{i}X^{i}\in T_{N}[X], \end{array}$$

where each coefficient $\mu_{i}\in \{0,\frac{1}{q}\}$ encodes one bit (Fig. 2). For a 9600-bit template with N=1024, the compression ratio is

$$\begin{array}{l} \frac{n+1}{k+1}\approx 270, \end{array}$$

achieving about 270× reduction over bitwise LWE encryption.

④  Encrypted templates together with BK and ksk are transmitted to DB and referenced by AS for later comparison. This reduces communication/storage costs while maintaining compatibility with homomorphic operations. Packed TLWE ciphertexts are converted to LWE via SampleExtract before gate-level evaluation, then re-packed only when needed.

### 4.3 Transformed message space for hamming distance

Biometric verification is modeled as binary comparison measured by the Hamming distance HD. ⑤  Both enrolled and query templates, stored in DB, are encrypted under the same K, while AS performs evaluation using BK and ksk.

*Modified Bootstrapped XOR*. Standard TFHE requires re-bootstrapping every XOR result; ToMAS redefines the LUT coefficient $\mu_{\text{LUT}}=\frac{1}{2q}$ and adds $\frac{1}{2q}$ to the output ciphertext, transforming the message space to $M^{\prime }=\{0,\frac{1}{q}\}$. This enables direct accumulation of XOR outputs without intermediate bootstrapping as shown in Algorithm 1.

Algorithm 1 **Modified bootstrapped XOR gate**

*Standard Bootstrapped XOR (for comparison)*. The standard bootstrapped XOR gate of TFHE is presented in Algorithm 2, which serves as the baseline for the modified XOR used in ToMAS.

Algorithm 2 **Standard bootstrapped XOR gate**

*Noise Control*. When L XORs are accumulated, noise grows linearly. ToMAS applies segmented summation: groups of size $L_{\text{seg}}$ (e.g., 32 or 64) are summed, bootstrapped, and hierarchically aggregated, keeping total variance

$$\begin{array}{l} \sigma_{\text{tot}}^{2}=\sigma_{0}^{2}\lceil \frac{L}{L_{\text{seg}}}\rceil \end{array}$$

below TFHE’s threshold even for L>1000.

#### 4.3.1 Secure hamming distance computation

⑥  The similarity between templates is evaluated as

$$\begin{array}{l} \mathrm{HD}(X,Y)=\sum_{i=0}^{L-1}(X_{i}⊕Y_{i}),\;{\mathrm{HD}}_{\text{norm}}=\frac{\mathrm{HD}(X,Y)}{L}. \end{array}$$

Each XOR output in $\left\{0,\frac{1}{q}\right\}$ can be directly accumulated, giving

$$\begin{array}{l} {\mathrm{ct}}_{\text{sum}}\approx \mathrm{Enc}\;\left(\frac{\mathrm{HD}(X,Y)}{q}\right). \end{array}$$

After two-step decryption (below), AS multiplies by q to obtain HD and divides by L if normalization is needed, as shown in Table 3.

**Table 3:** Execution time for Hamming distance evaluation.

Method	Phase	λ=80 (s)	λ=128 (s)
Modified Bootstrap	HE_XOR	0.059	0.063
	Summation	0.00065	0.00073
Original Bootstrap	HE_XOR	0.059	0.063
	Summation	7.081	8.247

Since XOR outputs live in $M^{\prime }=\{0,\frac{1}{q}\}$, the accumulator holds a torus-encoded sum; after SampleExtract to LWE, multiplying by q and rounding exactly recovers the integer HD.

## 4.4 Two-step decryption protocol

Decryption proceeds in two phases to protect the user key.

*Partial decryption by user* U. ⑦  AS sends a from $d_{\text{enc}}=(a,b)$ to U. ⑧  U computes $d^{*}=\langle a,K\rangle +e^{*}$, $e^{*}\;\leftarrow \chi$, and returns $d^{*}$ to AS.

*Final decryption by server* AS. ⑨   AS computes

$$\begin{array}{l} d_{\text{dec}}\equiv b-d^{*}\;(\mathrm{mod}\;1)=\frac{\mathrm{HD}}{q}+(e-e^{*})\in T. \end{array}$$

Then compute $\hat{\mathrm{HD}}=\text{round}(q\cdot d_{\text{dec}})$ and, if needed, ${\mathrm{HD}}_{\text{norm}}=\hat{\mathrm{HD}}/L$.

$$\begin{array}{l} \text{Decision: accept if}\;{\mathrm{HD}}_{\text{norm}}=\frac{\hat{\mathrm{HD}}}{L}\leq \tau . \end{array}$$

*Security*. Each $d^{*}$ contains fresh noise $e^{*}\;\leftarrow \chi$ and remains computationally indistinguishable under LWE; thus, even collecting multiple $d^{*}$ values yields no information about K or plaintexts.

## 5 Our secure biometric authentication system: ToMAS

### 5.1 System requirements

Building upon the cryptographic guarantees in Section 4, we retain the same four entities and roles already defined there. Here we restate only the operational assumptions relevant to the following analysis. The D is trusted for biometric capture and feature extraction during both enrollment and authentication; attacks on sensors or preprocessing modules are out of scope. All communication between D and AS is assumed to be authenticated and confidential, thereby mitigating tampering and man-in-the-middle attacks.

Under these assumptions, ToMAS guarantees that:

• AS reliably authenticates legitimate users.
• Communications between D and AS remain protected against active adversaries, including replay attempts.
• Biometric templates stored in DB stay encrypted and secure even if DB is compromised.

ToMAS implements multi-factor authentication by combining biometric traits with a password-derived secret key. All biometric data are encrypted before storage in DB, and both enrollment and authentication proceed entirely under homomorphic encryption. A timestamp-based replay defense preserves session integrity, while the authentication server performs only a keyless final recovery step on masked values, ensuring that no biometric or key material is ever exposed.

### 5.2 Overall description

Algorithm 3 outlines the end-to-end flow of ToMAS. Biometric templates are represented as binary vectors, ensuring compatibility with modalities such as iris, fingerprint, or vein recognition.

Algorithm 3 **End-to-end authentication in ToMAS**

## 6 Threat model and security analysis

We define the threat model considered in ToMAS and analyze how the system withstands various attacks. The analysis covers malicious users, passive adversaries, and active adversaries that attempt to compromise authentication or privacy. All cryptographic operations follow the guarantees in Section 4, under the trust boundaries of Section 5.

### 6.1 Threat model

We follow the same four entities and notation as defined in Section 4. ToMAS operates at the application layer and remains secure even if the underlying transport (e.g., TLS) is only partially protected. Encrypted templates and related ciphertexts are stored in DB and referenced by AS for verification.

We classify adversaries as follows:

• **Malicious user:** attempts to impersonate a legitimate U by submitting forged or altered biometric data.
• **Passive adversary:** gains unauthorized access to DB and observes encrypted biometric templates or intermediate ciphertexts.
• **Active adversary:** intercepts, modifies, or replays messages exchanged between D, DB, and AS.

### 6.2 Security against a malicious user

Even if an attacker attempts to impersonate a legitimate user, forged biometric data alone cannot succeed due to the uniqueness of biometric traits and the need for the password-derived secret key K. Without the correct PW, an adversary cannot regenerate K, making combined biometric–password forgery infeasible. Thus, ToMAS resists impersonation attacks at the device level.

### 6.3 Security against passive attacks

ToMAS inherits semantic security under chosen-plaintext attack (IND-CPA) from TFHE [20]. Even if an adversary fully compromises DB or AS, no information about the plaintext biometrics can be derived from ciphertexts. All encrypted values—including ${\mathrm{Enc}}_{K}(X)$, intermediate results $d_{\text{enc}}$, and partially decrypted values $d^{*}$— remain computationally indistinguishable from random under the LWE assumption. Hence, leakage from stored data or intercepted messages provides no advantage.

### 6.4 Security against active attack (Replay attack)

**Fig. 3:**
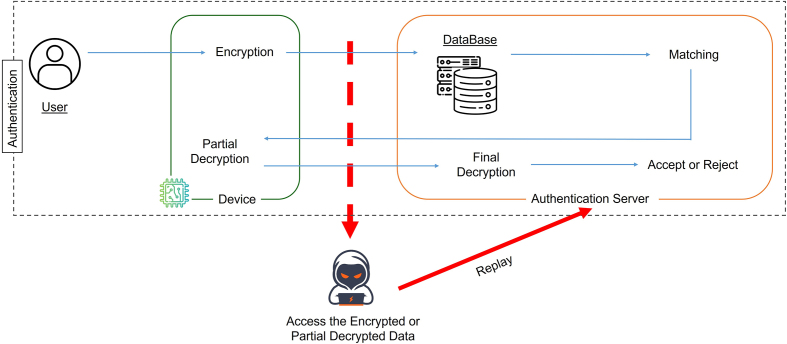
Illustration of a replay attack targeting DB–AS communication.

The most critical active threat in biometric authentication systems is the *replay attack*, in which an adversary resubmits previously captured authentication data. If a valid $\left(ID,{\mathrm{Enc}}_{K}(X)\right)$ pair from DB is replayed to AS, an attacker might trigger partial decryption and thereby bypass the authentication procedure (see Fig. 3).

**Fig. 4:**
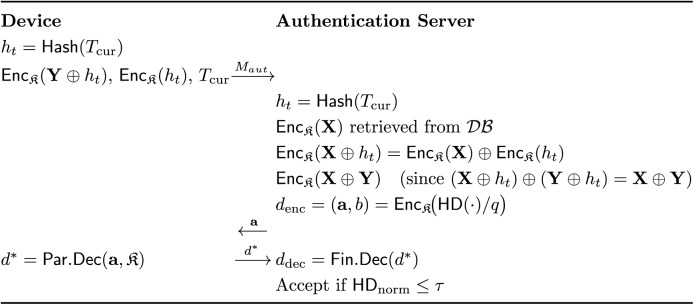
Overview of the timestamp-based anti-replay mechanism in ToMAS. All variable names match Algorithm 4.

ToMAS prevents such attacks by binding every authentication session to a unique timestamp $T_{\text{cur}}$. The D computes a session-specific mask $h_{t}=\mathrm{Hash}(T_{\text{cur}})$ and encrypts it together with the randomized query template: ${\mathrm{Enc}}_{K}(Y⊕h_{t})$ and ${\mathrm{Enc}}_{K}(h_{t})$. Upon receiving these, the authentication server AS retrieves the enrolled template ${\mathrm{Enc}}_{K}(X)$ from DB and computes

$$\begin{array}{l} {\mathrm{Enc}}_{K}(X⊕h_{t})={\mathrm{Enc}}_{K}(X)⊕{\mathrm{Enc}}_{K}(h_{t}), \end{array}$$

which is feasible under TFHE’s bitwise XOR operation since both ciphertexts share the same secret key K. Homomorphic evaluation of HD then proceeds on the masked ciphertexts. Because $h_{t}$ is valid only within that session, any replayed ciphertext or outdated timestamp results in mismatched randomization and inevitably fails authentication (Fig. 4).

Algorithm 4 **Timestamp-based Anti-Replay mechanism**

While transport-layer protocols such as TLS mitigate network-level replay or man-in-the-middle attacks, ToMAS enforces ciphertext-level replay resistance by session randomization. Even if encrypted templates are intercepted from DB or AS, timestamp-based masking invalidates any reused ciphertext. This layered protection complements TLS, ensuring end-to-end security for biometric authentication data.

## 7 Experimental evaluation

### 7.1 Experimental setup

We implemented ToMAS using the TFHE library on an AMD Ryzen Threadripper Pro (3.6 GHz, 64 GB RAM, Ubuntu 22.04). For empirical evaluation, we employed the *CASIA-IrisV1* benchmark dataset [46], which contains 756 high-resolution iris images from 108 individuals. Each image was preprocessed via normalization, quantization, and binarization to form a 9,600-bit iris code. Encrypted binary templates were stored in DB and referenced by AS during matching. U and D jointly handled encryption and transmission under the same security parameters. The full TFHE-based implementation is available at https://github.com/MiyeonHong/ToMAS.

### 7.2 Parameter setting via lattice estimator

The security of TFHE relies on the hardness of the LWE problem, which depends on the secret dimension n and the Gaussian error standard deviation σ. Table 4 lists three parameter sets corresponding to 80-, 112-, and 128-bit security, as determined by the lattice estimator [47]. Each configuration ensures that the estimated complexity of known lattice attacks exceeds $2^{\lambda }$ operations for the desired security level.

**Table 4:** TFHE parameter sets used in ToMAS.

Set	λ	LWE		TLWE			TGSW		ksk	
		n	σ	N	k	σ	$B_{g}$	ℓ	$B_{\text{ks}}$	$\ell_{\text{ks}}$
I	80	540	$2^{-20.2}$	1024	1	$2^{-31.6}$	64	3	16	5
II	112	600	$2^{-15.3}$	1024	1	$2^{-27.8}$	32	4	16	5
III	128	630	$2^{-13.8}$	1024	1	$2^{-24.1}$	64	3	32	4

The decomposition bases $B_{g}$ and $B_{\text{ks}}$, together with lengths ℓ and $\ell_{\text{ks}}$, were selected to balance efficiency and precision. This parameterization represents a practical trade-off between computational performance and cryptographic strength.

### 7.3 Performance evaluation

For each parameter set, we measured execution times of KeyGen, Enc, Dec, and HD computation, together with ciphertext sizes before and after packing. All measurements represent the average of 100 runs covering both enrollment and authentication. Ciphertext sizes were estimated by mapping torus elements T to 32-bit integers, as shown in Table 5.

**Table 5:** Performance of ToMAS at different security levels.

Set	Biometric (bits)	Time (s)				Ciphertext size	
		KeyGen	Enc	HD	Dec	Original	Packed
I	9600	0.937	0.0061	0.878	0.00032	20.77 MB	82 KB
II	9600	1.126	0.0065	0.941	0.00033	23.08 MB	82 KB
III	9600	1.635	0.0067	0.996	0.00035	24.23 MB	82 KB

Using TLWE packing (N=1024), 960 bits were packed per ciphertext, requiring 10 ciphertexts for a 9,600-bit template. This provided 253–295× storage reduction compared to bitwise LWE encryption. The HD evaluation was parallelized across L/N segments using 10 threads on AS. Under parameter set I, the mean HD evaluation time was 0.878 s.

### 7.4 Authentication accuracy

We evaluated genuine and impostor score distributions on plaintext *CASIA-IrisV1* templates. Because ToMAS computes the exact Hamming distance homomorphically, the decrypted results perfectly matched plaintext outcomes. The Equal Error Rate (EER) achieved was 0.83 % at threshold τ=0.334, where False Acceptance Rate (FAR) and False Rejection Rate (FRR) both equaled 0.83 %. The acceptance rule HD≤τ applies identically to both plaintext and encrypted evaluations.

### 7.5 Comparison with existing methods

Table 6 compares ToMAS with representative HE-based biometric authentication schemes. Metrics include template size, security level, ciphertext size, and Hamming distance computation time. All runtime data were taken from the corresponding papers; hardware differences are noted where applicable.

**Table 6:** Comparison of ToMAS with existing secure biometric authentication schemes.

Protocol	Scheme	Biometric (bits)	λ	Size (KB)	HD (s)	Client (s)	Server (s)
Kim et al. [48]	FH-IPE	750	112	132	2.000	0.753	2.700
Yasuda et al. [49]	Ideal Lattice (SHE)	2048	80	19	0.018	0.0199	0.0272
Torres et al. [50]	Lattice-based FHE	2048	100	517	415.8	229.8	442.2
GHOST [32]	SHE + MAC	2400	80	40.1	0.470	0.0212	0.375
Yang et al. [37]	Paillier (PHE)	600	–	–	3.000	252.0	3.028
Blind-Touch [42]	CKKS (DNN)	512 d*	128	–	∼0.2–0.3**	∼0.05**	∼0.2–0.3**
Blind-Match [43]	CKKS (cosine, 1:N)	128 d*	128	–	0.452	0.0296***	0.452
AMB-FHE [44]	CKKS (fusion)	1024 d*	128	–	–	–	–

**ToMAS (ours)**	TFHE	9600	80	82	0.878	0.0061	0.878
	TFHE	9600	112	82	0.941	0.0065	0.941
	TFHE	9600	128	82	0.996	0.0067	0.996

* CKKS-based works use feature vector dimension (d) instead of bit length.

** Blind-Touch reports real-time fingerprint inference (hundreds of ms).

*** Blind-Match client time denotes encryption only; HD time corresponds to server-side matching.

Recent CKKS-based schemes such as Blind-Touch [42], Blind-Match [43], and AMB-FHE [44] achieve real-time inference or multi-modal fusion but rely on approximate floating-point computations. In contrast, ToMAS performs exact bitwise comparison over 9,600-bit templates with sub-second latency and 80–128-bit security, while DB only stores encrypted vectors, eliminating any exposure of biometric or key material (K).

## 8 Discussion

This work has demonstrated the feasibility and practicality of applying Fully Homomorphic Encryption (FHE) to secure biometric authentication while preserving exact matching accuracy. By designing a two-phase protocol that performs both enrollment and authentication entirely in the encrypted domain, ToMAS provides privacy protection without compromising the cryptographic guarantees of TFHE. The proposed framework highlights how encrypted operations, including XOR-based Hamming distance evaluation and partial decryption, can achieve sub-second performance under realistic security parameters.

*Focused assumptions for core analysis*. Our security analysis deliberately excludes attacks targeting the sensor hardware and feature-extraction modules. This design choice was made to narrow the scope and concentrate on the homomorphic protection of the biometric template during the enrollment and authentication phases. Such a restriction enables rigorous formal reasoning about encrypted computation and its security properties without being confounded by non-cryptographic threats such as spoofing or presentation attacks. These assumptions align with prior FHE-based studies that also treat early-stage attacks as orthogonal to cryptographic mechanisms.

*Cryptographic reliability and system integrity*. Even within this constrained threat model, ToMAS ensures data confidentiality and integrity through its encryption pipeline. All biometric templates remain encrypted throughout their lifecycle, and the authentication server never learns any information about the plaintext or the user’s secret key (K). The combination of TFHE-based operations, partial decryption, and timestamped replay defense collectively guarantees semantic security under the LWE assumption. This layered protection reinforces the reliability of the proposed system even when communication channels are only partially secure.

*Future research directions*. While this study emphasizes the cryptographic aspects of FHE-based biometric authentication, several extensions remain promising. First, integrating FHE with secure hardware enclaves or trusted sensors could provide end-to-end protection, including the acquisition stage. Second, exploring efficient batching or SIMD-based TLWE packing may further accelerate encrypted template comparisons across multiple users. Finally, formal composability analysis—linking the FHE security model with higher-level authentication protocols—would enable a more holistic understanding of system-wide guarantees.

*Concluding remarks*. In summary, ToMAS establishes a solid foundation for privacy-preserving biometric authentication by showing that exact matching can be achieved under homomorphic encryption at practical speed. While our current focus remains on the cryptographic domain, future work will expand toward comprehensive, multi-layer defenses that unify cryptography, hardware, and biometric signal security. We view this research as a foundational step toward deployable biometric systems operating entirely under encrypted computation.

## 9 Conclusion

We presented **ToMAS**, a privacy-preserving biometric authentication framework that integrates multi-factor verification with TFHE-based homomorphic encryption. The proposed system performs all critical operations—including feature storage, encrypted Hamming distance computation, and decryption—entirely within the encrypted domain, thereby ensuring that no biometric or key material is ever exposed to the server.

By combining ciphertext packing with a modified bootstrapping mechanism for XOR aggregation, ToMAS achieves exact matching accuracy while significantly reducing computational overhead. Our evaluation on the *CASIA-IrisV1* dataset demonstrates sub-second encrypted authentication and practical performance under 80-, 112-, and 128-bit security parameters, surpassing prior HE-based schemes in both runtime and scalability. Furthermore, the integration of timestamp-based replay defense strengthens resilience against active adversaries without compromising efficiency.

In summary, ToMAS is practically deployable under standard constraints. Future research will extend this framework toward multi-modal biometric fusion and composable FHE protocols, providing end-to-end protection from data acquisition to decision making.

## Acknowledgments

This work was supported by Institute of Information & communications Technology Planning & Evaluation (IITP) grant funded by the Korea government(MSIT) (No.RS-2024-00460321, Development of Digital Asset Transaction Tracking Technology to Prevent Malicious Financial Conduct in the Digital Asset Market)

Ji Won Yoon is also partly supported by the fund from Korea University for the project, "Efficient Authentication Technology through De-identification Techniques."

## CRediT authorship contribution statement

**Mi Yeon Hong:** Writing – original draft, Visualization, Validation, Resources, Project administration, Methodology, Investigation, Funding acquisition, Formal analysis, Data curation, Conceptualization. **Kang Hoon Lee:** Visualization, Methodology. **Ji Hyuk Jung:** Formal analysis, Data curation. **Ji Won Yoon:** Writing – review & editing.

## Declaration of competing interest

The authors declare that they have no known competing financial interests or personal relationships that could have appeared to influence the work reported in this paper.
